# Improving Standards of Physical Health Care of Patients in Secure Mental Health Hospital

**DOI:** 10.1192/bjo.2022.327

**Published:** 2022-06-20

**Authors:** Harikrishna Patel, Kathryn Robinson, Alexandra Kennard

**Affiliations:** Southern Health NHS Foundation Trust, Southampton, United Kingdom

## Abstract

**Aims:**

Evidence suggests that individuals with serious mental illness (SMI) die up to twenty years prematurely compared to the average population without SMI with the main causes of death linked to preventable cardiovascular disease, respiratory failure, and endocrine disorders. This early mortality remains despite national efforts to recognise the issue and promote development of processes to enhance quality of physical health care in patients with SMI. The National Institute for Health and Care Excellence (NICE) clinical guidelines (CG178) recommendation 1.5.3.5 states that healthcare professionals in secondary care should ensure individuals with SMI receive physical health checks from primary care. This idea may be suitable for patients in the community setting or those who undergo shorter hospital admissions, however the process of psychiatric rehabilitation in secure mental health hospitals is challenged by long average stays resulting in no access to routine primary care facilities. The aim of the project is to introduce tailored measures that would aid in delivering high quality physical health care to patients within secure mental health hospitals.

**Methods:**

Ravenswood House Medium Secure Hospital supported a project to improve the physical health of individuals with SMI. An audit was completed to evaluate the assessment and management of baseline physical health measures that would have usually been completed in primary care as per the standards set out in NICE guidelines. The results showed that not all measures were being met and there was room for improvement.

Based on these NICE recommendations, an annual health check template and a centralised documentation toolkit were implemented and integrated within a new Physical Health Care Pathway in collaboration with General Practitioners, Dentists, Physiotherapists and other Allied Healthcare professionals.

**Results:**

Following implementation of the Physical Health Care Pathway, the number and quality of annual physical health checks in Ravenswood House Medium Secure Hospital increased resulting in significantly better-quality outcomes for patients by completing appropriate referrals and follow-up care.

**Conclusion:**

This collaborative approach of providing a high-quality physical health care was delivered in-house by arrangement with external healthcare practitioners. This pathway of providing care assisted us in overcoming several challenges faced within secure hospitals due to legal sanctions and related security protocols involving the patient group.

